# The So-Called Functional Symptoms in Organic Nerve Injuries

**Published:** 1918

**Authors:** 


***The So-Called Functional Symptoms in Organic Nerve Injuries.*** By J. S. B. Stopford. *The Lancet*, June 8, 1918, p. 795.

The author shows that patients with undoubted injury of a peripheral nerve, who also exhibit functional symptoms, may not only, as Babinski has suggested, have them produced by faulty treatment at the front; but also by medical suggestion, or as the author' suggests, by an emotional disturbance due to prolonged strain before being wounded.

He divides these mixed cases into two groups: (1) Cases in which the functional element predominates and where the case is called hysterical and where the organic defect is overlooked. (2) Cases in which the organic symptoms are more apparent. Here there are less chances of error in diagnosis in this group which is the predominant.

In patients with mixed symptoms thermalgesia occurs most frequently, and the author has uniformly found evidence of an irritative nerve lesion. Pain is a practically constant premonitory symptom of an irritation when it precedes the onset of the purely functional paralysis uncomplicated by external wounds. Unsatisfactory results of the treatment of the functional part of the syndrome are encountered if the irritative lesion was not rectified previously.

In practice it is found that the functional cases, which fail to react to treatment, or soon relapse, are the ones in which the organic part still persists. Trauma therefore plays a much less inconspicious part in the production locally, and in the persistance of functional manifestations, than it is usually considered to do and it appears to be the exciting cause, though not the only one.

There seems little doubt that the predisposing cause is of psychogenic origin, as exposure to the conditions of modern warfare is liable to promote psychic exhaustion to which strain some subjects will react more readily and more profoundly than others. Men who have previously exhibited definite hysterical symptom will react to this strain and are predisposed to suffer again from functional disturbance at an early date.

Toxaemia, insufficient nourishment, or loss of sleep, influences the reaction of an individual to psychic exhaustion and even under these circumstances the most stable might be thrown into a state predisposing to functional disturbance. Treatment must be rational and directed to all possible factors in the production of these conditions — i. e. the greatest patience and diligence must be shown in attempting to discover as a preliminary measure whether a source of irritation exists and if so, to treat it before treating the functional part. The psychic side is then easily relieved and then cured.

